# Understanding the Value of a Proactive Telecare System in Supporting Older Adults’ Independence at Home: Qualitative Interview Study Among Key Interest Groups

**DOI:** 10.2196/47997

**Published:** 2023-12-14

**Authors:** Lauren Fothergill, Carol Holland, Yvonne Latham, Niall Hayes

**Affiliations:** 1 Division of Health Research Faculty of Health and Medicine Lancaster United Kingdom; 2 Organisation Work and Technology Lancaster University Lancaster United Kingdom; 3 Leeds University Business School University of Leeds Leeds United Kingdom

**Keywords:** older adults, telecare, independent living, health and well-being

## Abstract

**Background:**

Telecare is claimed to support people to live in their own homes for longer by providing monitoring services that enable responses to emergencies at home. Although most telecare technologies commissioned in the United Kingdom predominantly supply reactive services, there has been recent interest among policy makers to develop proactive telecare services to provide additional understanding of older adults’ health and well-being needs to provide a means for more preventive interventions. Proactive telecare refers to providing regular well-being calls or encouraging users to regularly confirm their well-being to anticipate and prevent crises through an increased understanding of individuals’ needs and by building social relationships with older adults. Such technologies have already begun to be introduced, yet little research has explored the potential value of proactive telecare.

**Objective:**

This study explores the perceptions of different interest groups to understand the extent to which using a proactive telecare service can support older adults to live independently, what potential health and well-being benefits may be elicited from its use, and what the limitations are.

**Methods:**

Semistructured interviews were conducted with older people (those with experience in using proactive telecare and those without), family members of proactive telecare users, and proactive telecare staff regarding their perceptions and opinions about the value of a proactive telecare service. Data were analyzed using inductive thematic analysis.

**Results:**

A total of 30 individuals participated in this study. Older adults described the value of proactive telecare in feeling safe and in control and appreciated feeling connected. Family members and staff valued the potential to detect early health deterioration in older adults, and all participants highlighted the benefit of strengthening access to social networks, particularly for socially isolated older people. However, telecare is often viewed as a last resort, and therefore, anticipatory care may not suit all populations, as demonstrated by the mixed acceptance of the technology among older adults who did not have experience using it. Participants also reported limitations, including the requirement for family, friends, or neighbors to assist older adults during an emergency and the need for financial resources to fund the service.

**Conclusions:**

This study presents the first known qualitative inquiry about a proactive telecare system, which provides rich and detailed insights from different perspectives into the potential benefits of this intervention. Proactive telecare may promote and facilitate the accumulation of social and technological resources as individuals prepare to cope with age-related challenges, thus helping to avoid negative outcomes prematurely. However, similar to reactive telecare, proactive telecare must be matched to individual preferences and existing financial and social resources.

## Introduction

### Background

The United Kingdom faces an aging population. In 2018, approximately 1 in 5 people were aged ≥65 years, with this figure expected to reach 1 in every 4 people by 2038 [1]. In response, policy makers advocate for supporting older adults to live independently at home to avoid costly institutional care [2]. Studies suggest that maintaining independence is also a key desire of older adults [2,3] as it facilitates people aging well [4]. Independence-related concepts refer to maintaining autonomy, making choices [5], preserving physical and cognitive function, being self-reliant [6], and having the necessary financial and social resources to cope with age-related challenges [5]. Loss of independence contributes to reduced health-related quality of life [7], low self-esteem, depression, and feelings of worthlessness [8]; therefore, the public health benefits of promoting independence are substantial. However, interrelated factors threaten independence, such as physical and cognitive impairments, chronic diseases, and reduced social networks [9,10]. In response, technology use is encouraged in older adults to support and maintain independence [11].

Telecare is reported to have great potential in supporting people to live in their own home for longer. [12]. Telecare is characterized by monitoring technologies that manage the risks associated with independent living; examples include pendant alarms and fall detectors [13]. Telecare is typically connected to a call center, where assistance can be summoned, for example, if a person has fallen. Telecare is promoted by policy makers, who understand its potential in reducing hospital admissions and improving quality of life among older populations, and is routinely commissioned by most local authorities in England [14]. However, there is evidence suggesting that the uptake of telecare is relatively low [15], and researchers question its utility to support independence [16].

Most telecare services available are predominately reactive in nature. Reactive telecare refers to sensors or pendant alarms that trigger an emergency response following an alarm raised by the user or detection of unusual behavior by ambient sensors [17]. Reactive telecare has several limitations. First, pendant alarms are dependent on the individual to be activated during an emergency, which may not always be possible as devices may not be always worn [18], users may not be able to react, or they might delay reacting to a situation because they do not wish to inconvenience others [19]. Second, ambient sensors may manifest in concerns about being monitored, affecting perceived control and privacy [20]. Passive monitoring may shift agency away from the older person, providing little opportunity for user engagement and resulting in reduced autonomy [21]. Sanders et al [22] explored the barriers to adopting reactive telecare during the Whole Systems Demonstrator Program, a large evaluation of telecare effectiveness in England, and argued that older adults in their study associated reactive telecare with stigma and ageism. Telecare is, therefore, often viewed by older adults as a last resort [23,24], thus reducing its potential to promote health and well-being in later life.

The concept of proactive telecare has received interest among researchers [25,26] and policy makers [27]; however, research investigating such technology is still in its infancy, despite its existing use in countries such as Spain [17] and the United Kingdom [28]. Proactive telecare refers to providing proactive well-being calls or encouraging users to regularly confirm their well-being, with the aim of anticipating and preventing crises and facilitating strong social connections between older adults and social care services [17]. Having regular engagement with older adults may enable early identification of significant changes in needs [9], which could provide the user the opportunity to acquire resources to prolong independence or receive health care in a timely and preventive manner. Telecare that encourages active engagement from individuals to confirm their well-being, rather than using passive monitoring to detect ill-health, may elicit a sense of autonomy, which may support someone’s perceived goals of independence [29]. Proactively supporting older adults’ social care needs may act as an early warning system, which could provide a key mechanism to better assist older people to remain in their own homes; however, little research has explored its value in supporting independence.

### Objective

This study aimed to understand the extent to which using a proactive telecare service can support older adults to live independently, what potential health and well-being benefits may be elicited from its use, and what the limitations are. This qualitative study explored the perspectives of 4 key interest groups to gain an in-depth understanding of how proactive telecare may meet older adults’ independence needs, including older adults with or without experience of using proactive telecare, family members who support older adults to use the technology, or staff who deliver proactive telecare. Drawing on various experiences and perspectives from 4 participant groups ensured the collection of rich and candid data and maximized the potential of understanding the value, limitations, and outcomes of using a proactive telecare service.

## Methods

### The Proactive Telecare Service

**Figure 1:**
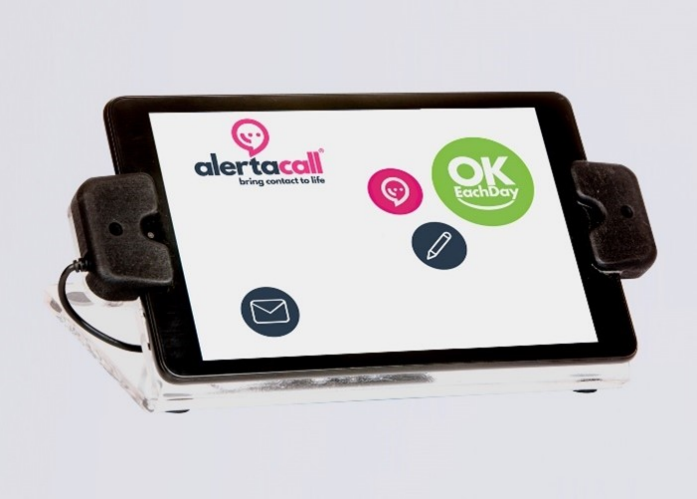
Proactive technology of interest (OKEachDay).

To gain insight into the potential value of a proactive telecare service in supporting independence, we explored the experiences and perceptions among key interest groups about an existing intervention in the United Kingdom. Housing providers are beginning to pilot proactive telecare services in the United Kingdom; however, there are few established proactive telecare services to use as examples. Therefore, for the purpose of this study, a service called OKEachDay was chosen owing to its known long-standing use across the United Kingdom. As the service had been operating since 2004, participants could reflect about their experiences of using the service over a long period, rather than restricted experiences during a pilot or trial. The technology consists of a touch screen smart device that is linked to a call center (Figure 1).

The technology requires older adults to confirm their well-being by pressing an “OK” button at an agreed time each day, either once a day or up to 3 times a day. If no contact is established, the call center team contacts the older adult to confirm their well-being, which gives the opportunity for information exchange or general social communication. If the older adult cannot be contacted, the call center team escalates the situation and contacts the user’s nominated contact, normally relatives, neighbors, or carers. This proactive telecare system provides support on a scale according to need, starting from a light touch service where older adults simply press the OK button once or several times in a day to a more involved intervention where older adults may not press their OK button and consequently receive further support from the proactive telecare staff. Call center staff are available from 8 AM to 10 PM every day of the year. Staff are given awareness training to provide low-level psychosocial support for older adults. Training includes topics about supporting emotional needs, mental health awareness, suicide awareness, discrimination and domestic abuse, dementia awareness, learning disabilities awareness, and safeguarding. Staff will signpost individuals if the issue goes beyond their knowledge or ability to help. The call center also conduct additional well-being calls to help people who may feel particularly isolated. The intervention is used in either sheltered housing, where the cost of the service is included in the independent living service charge paid by residents, or it is paid for privately by users. New users are sent the technology and given simple instructions about how it works either in person or over the phone. Staff contacts new users to explain how to use the system and answer any questions the users have. Systems are set up by plugging it into an electric socket, agreeing upon a convenient time for the user to press their OK button, and confirming the user’s nominated contact.

### Study Design

This study used semistructured interviews to conduct an in-depth exploration of different interest groups’ perspectives about the value of proactive telecare. Individuals from 4 groups were invited to participate to explore the phenomenon from different perspectives: (1) proactive telecare users; (2) family members of proactive telecare users; (3) proactive telecare staff involved in delivery; and (4) older adults who do not currently use proactive telecare, referred to as nonusers. The study design aligned with the COREQ (Consolidated Criteria for Reporting Qualitative Research) guidelines [30] (Multimedia Appendix 1).

### Ethical Considerations

Ethics approval for the study was given by Lancaster University ethics committee in June 2021 (FHMREC20142). Participants provided consent either verbally or via a consent form. All participants were offered a shopping voucher worth £15 (US $18.80) as appreciation for their time spent in the study.

### Participants, Recruitment, and Sampling

Participants were sampled purposively to ensure that perspectives from all identified interest groups were represented. Snowball sampling was also used to identify previously unknown participants [31]. The first author contacted managers at the proactive telecare organization and housing associations who use the proactive telecare service to aid in disseminating the study invitation. Current proactive telecare users and family members of users were invited to participate in the study via notification through the smart device or email. There were no relationships among the interviewees, that is, the recruited family members were not related to the recruited proactive telecare users. Older adults who did not currently use proactive telecare were recruited via local, older adult social groups. People who were interested in participating were sent a participant information sheet and asked to contact the first author. To meet the study’s inclusion criteria, older adult participants had to be aged ≥65 years and live in the community. If the participant wished to proceed, an interview time was agreed upon and consent was obtained.

### Data Collection

Data were collected between July 2021 and November 2021. Given the unpredictable nature of the COVID-19 pandemic at the time, interviews were conducted via telephone. A total of 30 semistructured interviews were conducted. Participants were assured that their participation was voluntary and were informed that they could withdraw at any stage. Interviews were conducted by the first author using an interview schedule. Interviews began by asking the participants about their views regarding independence, reasons for using proactive telecare, perceived health and well-being outcomes elicited, and limitations to use. Guides were adapted according to the specific interest group being interviewed (Multimedia Appendix 2). After 30 interviews, it was deemed that new data from the 4 groups were no longer adding further insights or dimensions to the overall findings, and therefore, through discussions, the researchers concluded that data saturation had been reached [32] and sufficient understanding of the emergent themes had been achieved. Interviews lasted between 25 and 80 minutes, with a mean time of 44 (SD 15.05) minutes. All interviews were audio recorded with permission from the participant and transcribed verbatim by the first author.

### Data Analysis

Data were analyzed following the stages of thematic analysis by Braun and Clarke [33], which provided a flexible, yet detailed and rich, analysis. The first author read the transcripts several times to facilitate immersion in the data. Inductive codes were recorded and grouped into potential candidate themes using NVivo (version 12; QSR International) software. Triangulating codes and themes from multiple interest group perspectives provided additional contextual information, which improved the interpretation of the data. To ensure credibility of the data analysis, initial codes and emerging themes were discussed with the senior research team, allowing further refinements. Codes were subsequently grouped into candidate themes and reviewed to ensure that data cohered together appropriately and meaningfully. Comparison of themes across interest groups enhanced the reliability and richness of the analysis [34]. Each theme was clarified, and meaningful names and descriptions were assigned. All researchers reviewed the final thematic outcomes.

## Results

### Participant Characteristics

In total, 30 participants were interviewed from various interest groups, comprising 15 (50%) proactive telecare users, 5 (17%) older adults who did not currently use proactive telecare, 4 (13%) family members of users, and 6 (20%) staff members (managerial and call center staff from the proactive telecare service and housing association staff who provide proactive telecare). The average age for the participant groups were as follows: proactive telecare users: 74.6 (range 65-87) years; older adults not currently using proactive telecare: 74.2 (range 67-81) years; staff involved in delivering proactive telecare: 39 (range 26-57) years; and family members of users: 65 (range 63-70) years. Overall, 3 (75%) out of 4 of the family members were female. Characteristics of the participants are displayed in Tables 1 and 2.

Overall, four themes were interpreted from the combined data: (1) health and safety, (2) autonomy, (3) access to social networks, and (4) needs and resources.

**Table 1:** Characteristics of proactive telecare users and older adults who are not currently using proactive telecare (nonusers).

Characteristics			Proactive telecare users (n=15), n (%)		Nonusers (n=5), n (%)
**Sex**					
	Male	7 (47)		1 (20)	
	Female	8 (53)		4 (80)	
**Level of care**					
	Informal or formal care	6 (40)		0 (0)	
	No care	9 (60)		5 (100)	
**Level of mobility**					
	Partially affected or limited	9 (60)		1 (20)	
	No issues	6 (40)		4 (80)	
**Employment status**					
	Retired	14 (93)		5 (100)	
	Employed part time	1 (7)		0 (0)	
**Current or previous occupation**					
	Professional	1 (7)		4 (80)	
	Managerial	2 (13)		0 (0)	
	Clerical and service	6 (40)		0 (0)	
	Trade work	4 (27)		0 (0)	
	Unemployed	1 (7)		1 (20)	
	Prefer not to say	1 (7)		0 (0)	
**Living arrangements^a^**					
	Private accommodation	10 (67)		4 (80)	
	Housing association	5 (33)		1 (20)	

^a^All older adults (20/20, 100%) lived alone.

**Table 2:** Characteristics of the staff involved in proactive telecare delivery (n=6).

Characteristics			Staff involved in proactive telecare delivery, n (%)
**Organization**			
	Housing association	3 (50)	
	Proactive telecare service (managerial and call center staff)	3 (50)	
**Sex**			
	Male	1 (17)	
	Female	5 (83)	

### Theme 1: Health and Safety

#### Feeling Safe and in Control

All participants acknowledged the priority of older adults to live in their own home; however, a key concern across participants was safety. Approximately half of the proactive telecare users had experienced a stressful event that influenced their independence, including onset of illness, loss of partner, or previous experience of falling, which subsequently led users to adopt proactive telecare. The remaining half of older adults anticipated age-related losses and adopted proactive telecare as a risk management strategy. Although the nonuser participants had not experienced a stressful event, all expressed fears of becoming dependent.

The proactive nature was viewed positively by most users and seemed to give both older adults and family members peace of mind that emergency action was not dependent on the user summoning help:

Well mentally, I think it helps anyway. Because otherwise you’d be worried all the time so mentally it’s a very good thing.

Proactive telecare user 12

Of the 5 nonusers, 2 felt that a proactive check-in may help to provide them with a network of social support that could help in case of future age-related deterioration, as the participant lacked close relatives:

If I had one of those [proactive telecare], it would relieve some of my anxieties that I have when I wake up at 5am in the morning every morning, one of the things that if you don’t have family around, you worry about. So, I do think OK, what do I do in the future and how do I organise ahead for this, is that something I may need as I get older, rather than waiting like my parents.

Nonuser 2

Daily check-ins were particularly important to some users who felt that they lacked social contacts who check in on them regularly. In contrast, some users did not wish family members to check on them physically and viewed the technology as a proactive check-in, which elicited feelings of self-efficacy. Some proactive telecare staff and users reported that when users start using the service, they required a few weeks to develop a routine of pressing their OK button:

When we first install, most of them forget for a couple of weeks. We have a big embedding period where for two weeks we just will call them, it just takes a bit to get into the swing of the routine.

Staff member 4; managerial proactive telecare staff

Once a routine was established, all users found the technology easy to use and were reassured that help could be accessed.

#### Limits to the Safety Element

For some participants, particularly nonusers, the fact that proactive telecare did not provide 24-hour support was a significant limitation, given that many proactive telecare users adopted the intervention to use as a safety precaution. Some users used pendant alarms alongside proactive telecare to solve this issue; however, acceptance toward the pendant alarm was mixed:

The pendant alarm is OK if there’s any emergencies. I mean two hours and 10 hours on the floor is a long time if you’re not very well. So, I keep the pendant around my neck in case I need any help.

Proactive telecare user 11

Some users considered proactive telecare as a precursor technology to other monitoring technologies such as ambient sensors, as they perceived themselves as independent and viewed monitoring technologies as intrusive and disempowering.

#### Identifying Health Deterioration

According to a few family members and staff members, proactive telecare had the potential to detect health deterioration in the user. These participants postulated that a lack of promptness of pressing the “OK” button over sustained periods may enable the detection of illness:

There have been significant periods where she had forgotten to press and it’s also always coincided with a period of when she hasn’t been so well, so I think it’s a good indicator.

Family member 2

This was considered as a significant benefit as family members suggested that older adults can find it difficult to ask for help, resulting in ill-health going undetected and consistent anxiety among some family members regarding older relatives’ health.

### Theme 2: Autonomy

Maintaining autonomy was a key priority expressed by many older adults and was associated with positive well-being. Despite experiencing physical decline, approximately half of the older adults were highly determined to do things by themselves, even if this required overexerting themselves. Other older adults, however, were more willing to forfeit some control and receive help from others as a compensation for loss of physical function, so that they could remain living at home.

For proactive telecare users, proactively pressing a button evoked a sense of agency and autonomy. Staff members at housing associations saw the benefits of giving control to users, as it demonstrated that they were viewed as capable to be responsible for their own well-being, potentially boosting confidence and self-esteem:

I think there’s a lot of benefits to it. It gives you a sense of freedom, it gives you independence, because you’re in charge of doing that.

Proactive telecare user 14

A user spoke about how they were offered a daily call instead of pressing a button, but they wanted to continue engaging proactively, as it enabled their independence and sense of capability. However, of the nonusers still regarded proactive telecare use as signifying older age:

Friends of mine who have disabilities would not use services like this...because they wouldn’t see themselves within the community of people who need them. I think there’s an issue around people not identifying themselves as being part of the group of people who require this support.

Nonuser 2

### Theme 3: Access to Social Networks

#### Opportunity for Social Connectedness

The potential for social support was viewed favorably across the participant groups, as it was perceived to provide an avenue of communication, particularly for individuals who struggle to ask for help from close contacts. Proactive telecare staff members viewed the call center as a valuable opportunity to check in with an individual’s well-being:

We have people call us that are suicidal, and that’s actually quite common now, we’re seeing that more and more...people just calling for help, they don’t know who else to call.

Staff member 4; managerial proactive telecare staff

Nonusers acknowledged the benefit of the social connection that the technology gave to people and saw this as an accessible way for someone feeling isolated to reach out and talk to someone. Almost all older adults appreciated having the option to call somebody, as it created another contact to call for help, separate from family and friends, where some older adults voiced concerns of being a burden.

#### Connections With the Staff

Discussions with older adults highlighted the importance of the relationships built with the call center staff. Older adults commonly mentioned the altruistic nature of the call center staff as beneficial, as this created a sense of belonging and reduced the feelings of loneliness:

If I were really lonely, and I were feeling down, I could phone somebody at [proactive telecare] and talk to them, cos the lady who usually phones me when I’ve missed the button, she’s very, very nice.

Proactive telecare user 8

However, it was acknowledged by some staff members that relationships between staff and users can take time to form and that individuals may not benefit from connections to this social network immediately or at all, if they do not want to engage with the social aspect of the technology.

#### Feelings of Burden

For some users, forgetting to press their button and receiving a call from the call center brought feelings of shame and embarrassment for being forgetful:

When they ring me, they’re very nice, but I feel like I’ve let myself down of forgetting to press the button.

Proactive telecare user 10

According to some older adults, forgetting to press their button was felt as being a threat to their perceived identity of being independent, as they wanted to be seen as able to cope by others. A few users spoke about feeling like a burden for forgetting to press their button, as they feared that the call center staff would be worried about their well-being.

### Theme 4: Needs and Resources

#### Perceived Need and Acceptance

Approximately half of the participants suggested that for people to adopt and benefit from the technology, they needed to have a level of acceptance regarding their age and related physical deterioration. Most proactive telecare users were future-orientated people and wanted to plan for anticipated age-related deficits but recognized that not all individuals have this mindset and, therefore, would not benefit from being proactive:

If you’re getting older, you don’t like to admit it. You still think you can do everything, until something happens.

Proactive telecare user 2

In contrast, 2 of the 5 nonusers acknowledged that they would not want to identify themselves as requiring support to live independently and would not consider using proactive telecare.

#### Reliance on Existing Networks

Approximately half of the participants voiced the concern that existing social networks were required for proactive telecare to be effective in providing safety. A nonuser highlighted that certain people who are socially isolated may struggle to give an emergency contact, and therefore, this type of intervention may not be appropriate. In addition, for most family members, it was important to be geographically close to their relative, so that they could provide support:

It would be more worrying if people were much further away, I would think, maybe it’s not the right system for them. Because first port of call really needs to be someone within easy reach or easy getting to the person that hasn’t pressed the button.

Family member 1

The remaining half of participants did not mention the need for social networks, but most of these users had relatives or friends close by and may not have realized this reliance. A few users mentioned feelings of uncertainty and anxiety in anticipation of an emergency, as their contacts did not live close. Some users and a family member had purposively established relationships with neighbors, to use them as a primary contact during an emergency; however, participants acknowledged this may not always be possible.

#### Financial Resources

Financing the intervention was seen as a key barrier to access by most. It was acknowledged that the financial commitment required may prevent older people from being proactive, and they may engage with it only after it becomes a necessity:

Well, it’s not free, is it, that’s the thing. And until you need it [proactive telecare], I guess you don’t realise it’s important...and I think a lot of people probably put it off.

Family member 4

In housing associations, proactive telecare was included in the package of living in the accommodation, which was seen by staff and users as a significant benefit and reduced economic barriers to access.

## Discussion

### Principal Findings

This study collected data from various interest groups to understand the extent to which a proactive telecare service could support independent living in older adults. Overall, our findings demonstrate benefits that overlap with those of reactive telecare, such as contributing to feelings of safety and providing reassurance of assistance in times of need. Nevertheless, this proactive telecare service presented unique benefits and challenges worth discussing. Giving the user the opportunity to confirm their well-being proactively facilitated autonomy and generated data with the potential to identify early health deterioration. Moreover, well-being calls presented the telecare staff with the opportunity to engage meaningfully with vulnerable service users and offered an additional source of social connection. However, our study suggested that forgetting to engage with proactive telecare may elicit feelings of burden, and individuals may have varying levels of social and financial resources, which must be assessed to ensure that older adults are best supported.

The desire to feel safe at home has been previously cited as a core motivation for adopting telecare [35], as older adults are more likely to be exposed to risks threatening their independence [36]. Reactive telecare is often used *after* an age-related incident and, subsequently, can symbolize negative stereotypes associated with aging [19]. In contrast, in this study, approximately half of the older adults adopted proactive telecare *before* they had experienced an age-related stressor but anticipated this risk and, therefore, saw the value in planning for the future. In addition, 2 of the 5 nonusers were concerned about the anticipated age-related challenges and did not want to age at home without adequate technological provisions. The preventive and corrective proactivity model describes the value of proactive adaptions in both anticipation of and in response to age-related changes, to accumulate resources to avoid and ameliorate the adverse effects of stressors [37,38]. Proactive telecare services that encourage uptake before age-related issues arise may facilitate the accumulation of social and technological resources to ensure safety at home, as individuals prepare to confront and cope with age-related challenges, thus helping to maintain well-being and quality of life [37]. However, some nonusers still associated proactive telecare with the stigma of aging; therefore, it is acknowledged that not all older adults may be receptive to adopting telecare before they perceive a need for it [39]. Our findings emphasize the need to offer a variety of interventions to suit different coping styles, which, in turn, may improve access to telecare and serve a wide population of older adults.

Recently, interest has grown in using proactive telecare to track patterns of behavior to monitor health in the home environment [25,40]. In this study, family members and housing association staff reported that tracking forgetfulness patterns of when a user had forgotten to press their OK button may help to detect early health deterioration, such as a urinary tract infection, which can cause confusion quickly [41] and may present as an individual forgetting to press their button over a short period. Detecting the early indicators of illness may offer the potential to inform early and more tailored interventions to support well-being and resilience [25] and avoid age-related stressors [17]. In contrast, tracking forgetfulness patterns may also provoke anxiety in older adults [42] and diminish well-being owing to the stigma associated with memory loss [43], as demonstrated in our data by the dismay expressed by users when they forgot to press the button. Our findings contribute new knowledge about the potential benefits and unintended consequences of proactive engagement with telecare and emphasize the need for further studies into the psychological implications of forgetfulness tracking.

Most older adults maintained the desire to sustain autonomy, which was associated with well-being [5,20]. In this study, older adults reported feeling self-sufficient by engaging proactively with the technology. Being self-reliant may bolster self-esteem and subsequently increase perceived sense of control [44], which is associated with better physical and psychological health [45]. Reactive telecare has focused on surveillance, which treats older adults as passive recipients of care and reduces their sense of control over technological use [46]. In contrast, our findings highlight the potential of proactive telecare in promoting self-management and, subsequently, independence, rather than conveying the need to be continuously monitored, thus signifying the value of proactive technologies in supporting independence. Notably, this proactive telecare system provided support according to the user’s level of independence, that is, if an older adult required further support, this was detected by the individual not pressing their OK button, or if an older adult pressed their OK button, it is assumed that no additional support was required. As the system monitored individuals according to their level of independence, this proactive approach may aid in ensuring that the right level of support is available during times when independence levels may change, for example, during periods of illness or following a hospital discharge. Further studies are required to understand the extent to which proactive telecare can detect the changing levels of independence across individuals and the potential benefits to health and well-being. Moreover, further studies are needed to understand the value of proactive telecare for individuals with high levels of cognitive dependency, as none of the participants in this study were living with dementia.

Participants saw social connections as important in maintaining independence [47]. Recent studies suggest that delivering outbound calls to older adults may foster strong relationships between users and service providers, helping to identify changes in people’s circumstances and provide more tailored support [17]. Similarly, in our study, proactive telecare users appreciated being connected to a network of support and valued the opportunity to disclose well-being issues, such as anxiety and loneliness. Gradual deterioration in mobility that accompanies aging may expose older people to social disconnection and loss of key social relationships [48], further highlighting the need to provide additional avenues of support to this population. There were limitations to proactive telecare. Some participants reported concerns regarding safety, as this service did not provide 24-hour support. Interestingly, some users had accepted this limitation as they saw other reactive telecare services as a symbol of older age, whereas other users chose to use additional technologies such as a pendant alarm for obtaining help faster in a crisis, thereby demonstrating the importance of assessing a person’s needs to best allocate telecare devices [49]. Another limitation was the reliance on the availability of family, friends, or neighbors to assist during a time of need, a limitation that also exists in reactive telecare [50]. This further emphasizes that telecare devices are not “one” solution but should work to complement people’s needs and resources [21]. Financial resources have also been identified as key barriers to telecare access [51]. However, little has been suggested to overcome these barriers. In this study, respondents commented about the benefit of having proactive telecare included in the package of living in sheltered accommodation, which relieved the burden of financial stress, thus demonstrating the potential advantages of package telecare systems.

### Limitations of the Study

This study has several limitations worth noting, particularly regarding the transferability of the findings. It is recognized that some participants may have expressed more positive views toward the intervention as a long-standing recipient or staff member, despite the interviewer asking participants to reflect about both positive and negative experiences. Although nonusers were invited to provide an outsider’s perspective about the technology and to give critical insights, these participants were recruited through an opt in method and may be more socially engaged, and therefore, their views may not be representative of this age group. All participants (30/30, 100%) were White British, and thus, these findings may lack transferability across different ethnic groups. Furthermore, this study only explored the experiences and opinions of 1 proactive telecare system; however, studies of proactive telecare are still scarce. Therefore, these findings may provide useful insights to direct further studies.

### Conclusions

This paper presents the first known qualitative inquiry about a proactive telecare system and provides insights into how this type of telecare may support older adults to live independently. Engaging proactively with telecare provides older adults access to social networks and support, if required. Having control over engagement with the technology helped bolster individual confidence and self-reliance, thus supporting independence and well-being. Daily engagement with technology offered opportunities for families to detect early health and well-being deterioration. As with other telecare, individual preferences and social and financial resources must be considered to maximize benefits.

## Appendix

Multimedia Appendix 1 COREQ (Consolidated Criteria for Reporting Qualitative Research) checklist.

Multimedia Appendix 2 Interview guides.

## Abbreviations

COREQ: Consolidated Criteria for Reporting Qualitative Research
