# Supplementary material for: Understanding the Value of a Proactive Telecare System in Supporting Older Adults’ Independence at Home: Qualitative Interview Study Among Key Interest Groups
Source: J Med Internet Res. 2023 Dec 14;25:e47997. doi: 10.2196/47997 (PMC10732490; doi:10.2196/47997)
Supplement: Multimedia Appendix 1 [file jmir_v25i1e47997_app1.pdf]

Supplementary file 1. Checklist for adherence to the consolidated criteria for reporting qualitative studies (COREQ)

Topic and Item No.	Guide Questions/Description	Response
**Domain 1: Research team and reflexivity**		
Personal Characteristics		
	1. Interviewer/facilitator: Which author/s conducted the interviews?	Lauren Fothergill (LF)
	2. Credentials: What were the researcher’s credentials? E.g. PhD, MD	LF: MPH
	3. Occupation: What was their occupation at the time of the study?	LF: Research Assistant/ PhD student
	4. Gender: Was the researcher male or female?	Female
	5. Experience and training: What experience or training did the researcher have?	LF: Mixed-methods researcher, experienced interviewer, GCP trained.
Relationship with participants		
	6. Relationship established: Was a relationship established prior to study commencement?	Researcher met the participants during recruitment
	7. Participant knowledge of the interviewer: What did the participants know about the researcher? e.g. personal goals, reasons for doing the research	Participants knew that LF was a researcher. They knew that the researcher was affiliated to the participating university.
	8. Interviewer characteristics: What characteristics were reported about the interviewer/facilitator? e.g. Bias, assumptions, reasons and interests in the research topic	Participants knew that the researcher were interested in their perspectives of the utility of using proactive telecare and to what extent it could support independence.
**Domain 2: Study design**		
Theoretical framework		
	9. Methodological orientation and Theory: What methodological orientation was stated to underpin the study? e.g. grounded theory, discourse analysis, ethnography, phenomenology, content analysis	Thematic analysis
Participant selection		
	10. Sampling: How were participants selected? e.g.	Purposive (e.g. for older adults – they were using

	purposive, convenience, consecutive, snowball	proactive telecare, family members – they had a relative using proactive telecare, staff – they were involved in delivering proactive telecare, older adults not currently using telecare – recruited from local older adult social groups).
	11. Method of approach: How were participants approached? e.g. face-to-face, telephone, mail, email	Participants were approached and recruited via telephone.
	12. Sample size : How many participants were in the study?	30 semi-structured interviews were conducted across various participant groups; (20 older adults, 4 family members and 6 staff members).
	13. Non-participation: How many people refused to participate or dropped out? Reasons?	Three people showed interest in the study but were not able to attend due to other commitments.
Setting		
	14. Setting of data collection: Where was the data collected? e.g. home, clinic, workplace	Data were collected over the phone.
	15. Presence of non-participants; Was anyone else present besides the participants and researchers?	No.
	16. Description of sample: What are the important characteristics of the sample? e.g. demographic data, date	Older adults: age, gender, level of care, level of mobility, living arrangements, ethnicity, previous occupation. Staff: organisation, age, gender. Family member: age, gender, age of family member using proactive telecare.
Data collection		
	17. Interview guide: Were questions, prompts, guides provided by the authors? Was it pilot tested?	No

	18. Repeat interviews: Were repeat interviews carried out? If yes, how many?	No
	19. Audio/visual recording; Did the research use audio or visual recording to collect the data?	Interviews were audio-recorded using a recording device.
	20. Field notes: Were field notes made during and/or after the interview or focus group?	Yes.
	21. Duration: What was the duration of the interviews or focus group?	Approx. 44 minutes (range from 25-80 minutes).
	22. Data saturation: Was data saturation discussed?	Yes.
	23. Transcripts returned: Were transcripts returned to participants for comment and/or correction?	No
**Domain 3: analysis and findings**		
Data analysis		
	24. Number of data coders: How many data coders coded the data?	One researcher was involved in coding, however, the themes were discussed between three researchers until themes were finalised.
	25. Description of the coding tree: Did authors provide a description of the coding tree?	No, however initial coding was informed by the interview guide, and coding was continuously refined.
	26. Derivation of themes: Were themes identified in advance or derived from the data?	Themes were derived from the data.
	27. Software: What software, if applicable, was used to manage the data?	NVivo 12
	28. Participant checking: Did participants provide feedback on the findings?	No
Reporting		
	29. Quotations presented: Were participant quotations presented to illustrate the themes / findings? Was each quotation identified? e.g. participant number	Yes
	30. Data and findings consistent: Was there consistency between the data presented and the findings?	Yes

	31. Clarity of major themes: Were major themes clearly presented in the findings?	Yes
	32. Clarity of minor themes: Is there a description of diverse cases or discussion of minor themes?	Yes
