# Supplementary material for: Understanding the Value of a Proactive Telecare System in Supporting Older Adults’ Independence at Home: Qualitative Interview Study Among Key Interest Groups
Source: J Med Internet Res. 2023 Dec 14;25:e47997. doi: 10.2196/47997 (PMC10732490; doi:10.2196/47997)
Supplement: Multimedia Appendix 2 [file jmir_v25i1e47997_app2.pdf]

Supplementary File 2: Interview guides

Questions for proactive telecare users

Experience and perception of being independent

Firstly, I'd like to talk to you about your experience and views on living independently at home.

What does living independently mean to you?

- How would you describe your level of independence?

Do you think there are any benefits to living independently? If so, what are they?

- Do you think there are any health benefits to living independently? Mental health, physical health, quality of life?

Do you think there are any negatives to living independently? If so. What are they?

- Any negative health impacts to living independently? Mental health/loneliness, physical health, quality of life.

Experience and views on using proactive telecare

Next, I'd like to talk to you about your experience and views on using proactive telecare

When did you get proactive telecare? Why did you get it?

How long did it take to adapt to using OK each day? How do you feel about using it?

- Did it take long to figure out how it worked? Did you need help to understand how to use it?

Why do you continue to use proactive telecare?

What do you like about proactive telecare?

- What benefits do you think there are to using it? Health benefits?

What don't you like about proactive telecare?

- Do you think there are negatives to using it? If so, what are they? Can you see any unintended consequences to using proactive telecare? Negative health impacts?

Have you noticed any changes in life since using proactive telecare?

Who do you think could benefit/or not benefit from using this (people with mild dementia, people living on their own, vulnerable people)?

Demographic questions

Lastly, I have some demographic questions for you. Can you please tell me:

How old are you?

How would you describe your gender?

- Male
- Female
- Other (please specify)
- Prefer not to say

How would you describe your ethnic background?

- White
- Mixed/multiple ethnic group
- Asian/Asian British
- Black/African/Caribbean/black British
- Other
- Prefer not to say

How would you describe your current/previous employment status?

- In full-time paid work
- In part-time paid work
- In full-time education
- In part-time education
- Full-time carer/homemaker
- On leave/out of work due to illness or disability
- Retired
- Other, please specify
- Prefer not to say

What are your current living arrangements?

- Live alone
- Live with partner/spouse/children
- Live with other adults
- Other, please specify

In general, would you say that your level of mobility is

- Partially affected/limited
- No issues

Do you have any formal/informal care?

Questions for family members of proactive telecare users

Experience and perception of independence for their family member

Firstly, I'd like to talk to you about your experience and views on your relations independence at home.

What does living independently mean to you and your family?

- How would you describe your family members/friends level of independence?

Do you think there are any benefits to living independently? If so, what are they?

- Do you think there are any health benefits to living independently? Mental health, physical health, quality of life?

Do you think there are any negatives to living independently? If so. What are they?

- Any negative health impacts to living independently? Mental health/loneliness, physical health, quality of life.

Views on their relation using proactive telecare

Next, I'd like to talk to you about your experience and views of your relation using proactive telecare

When did your family member/friend get proactive telecare? Why did they get it?

How long did it take for them adapt to using proactive telecare? How do you feel about the tool?

- Did it take long to figure out how it worked? Did they need help to understand how to use it?

Why does your family/friend you use proactive telecare? How does it affect you? (Safety, wellbeing, peace of mind?)

What do you like about proactive telecare? What benefits do you think there are to using it? health benefits, well-being?

What don't you like about proactive telecare? Do you think there are negatives to using it? If so, what are they? Can you see any unintended consequences to using proactive telecare? Negative health impacts?

Have you noticed any changes in life since your relation started using proactive telecare?

Who do you think could benefit from using proactive telecare (people with mild dementia, people living on their own, vulnerable people?

Demographic questions

Lastly, I have some demographic questions for you. Can you please tell me:

How old are you?

How would you describe your gender?

- Male
- Female
- Other (please specify)

- Prefer not to say

Questions for proactive telecare staff

Demographic questions

I have some demographic questions for you. Can you please tell me:

What organisation do you work for and what is your role?

How old are you?

How would you describe your gender?

- Male
- Female
- Other (please specify)
- Prefer not to say

Experience and perception of independence for older adults

Firstly, I'd like to talk to you about your views on older adults independence in the home environment

What does living independently mean to you and the organisation?

Do you think there are any benefits to living independently? If so, what are they?

- Do you think there are any health benefits to living independently? Mental health, physical health, quality of life?

Do you think there are any negatives to living independently? If so. What are they?

- Any negative health impacts to living independently? Mental health/loneliness, physical health, quality of life.

Views on proactive telecare

Next, I'd like to talk to you about your experience and views of delivering proactive telecare

Describe your experience of using proactive telecare in your role /tell me about your role in delivering proactive telecare.

(housing association staff) Why do you think [organisation name] use proactive telecare?

What do you like about proactive telecare? What benefits do you think there are to using it for older people?

- Health benefits to residents/organisational benefits

What don't you like about proactive telecare? Do you think there are negatives to using it for older people? If so, what are they?

- Negative health impacts to residents/organisational impacts?

(housing association staff) Have you noticed any changes in resident's lives since using proactive telecare? Any changes in the organisation?

Who do you think could benefit from using proactive telecare (people with mild dementia, people living on their own, vulnerable people?

Questions for older adults who currently do not use proactive telecare

Experience and perception of being independent

Firstly, I'd like to talk to you about your experience and views on living independently at home.

What does living independently mean to you?

- How would you describe your level of independence?

Do you think there are any benefits to living independently? If so, what are they?

- Do you think there are any health benefits to living independently? Mental health, physical health, quality of life?

Do you think there are any negatives to living independently? If so. What are they?

- Any negative health impacts to living independently? Mental health/loneliness, physical health, quality of life.

Views on using proactive telecare

Next, I'd like to talk to you about your views on proactive telecare service

Would you use it? And why? How would you feel about using it? Could you see yourself using it daily?

Who do you think could benefit from using this proactive telecare service (people with mild dementia, people living on their own, vulnerable people)?

What do you like about this proactive telecare service? Do you think there could be benefits to using it? If so, what are they?

- Health benefits?

What don't you like about this proactive telecare? Do you think there could be negatives to using it? If so, what are they? Can you see any unintended consequences to using proactive telecare service?

- Negative health impacts?

You can choose how often you press the button, once a day, twice a day, what do you think of this?

- You could have a phone call everyday instead of pressing the button, what do you think of this?/who do you think this might benefit? Having someone to check in has been viewed as an opportunity to have a chat if you want, is this something you can see yourself doing? If so, why?
- The system can also send reminders if you want, so say for doctor appointments, picking up prescription, would you find this useful or not? Who could this be useful for?

Demographic questions

Lastly, I have some demographic questions for you. Can you please tell me:

How old are you?

How would you describe your gender?

- Male
- Female
- Other (please specify)
- Prefer not to say

How would you describe your ethnic background?

- White
- Mixed/multiple ethnic group
- Asian/Asian British
- Black/African/Caribbean/black British
- Other
- Prefer not to say

How would you describe your current/previous employment status?

- In full-time paid work
- In part-time paid work
- In full-time education
- In part-time education
- Full-time carer/homemaker
- On leave/out of work due to illness or disability
- Retired
- Other, please specify
- Prefer not to say

What are your current living arrangements?

- Live alone
- Live with partner/spouse/children
- Live with other adults

- Other, please specify

In general, would you say that your level of mobility is

- Partially affected/limited
- No issues

Do you have any formal/informal care?
